# Supplementary material for: PDX Models: A Versatile Tool for Studying the Role of Myeloid-Derived Suppressor Cells in Breast Cancer
Source: Cancers (Basel). 2022 Dec 13;14(24):6153. doi: 10.3390/cancers14246153 (PMC9777315; doi:10.3390/cancers14246153)
Supplement: Supplementary file 1 [file cancers-14-06153-s001.zip › cancers-2092262-supplementary.pdf]

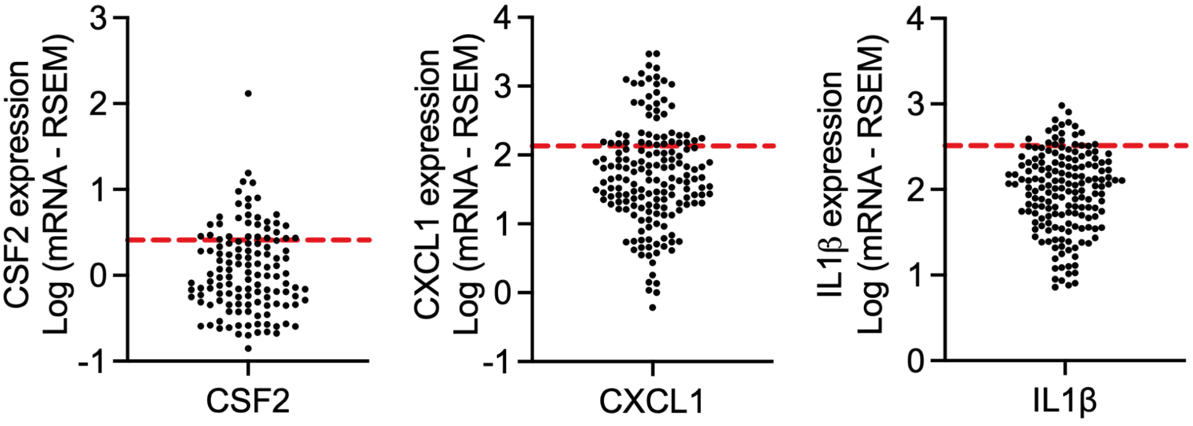

Supplementary figure S1. *Cytokine expression in patients with basal-like breast cancers. mRNA expression of CSF2 (n=130), CXCL1 (n=171) and IL1b (n=171) in patients with basal-like breast cancers using data from a cohort of breast invasive carcinomas of TCGA, PanCancer Atlas. High expression was defined as values 3x above the cohort median expression value as indicated by the red dotted line. The percentage of patient with high expression was IL1b=11,6%, CXCL1=27% and CSF2=17,6%.*

Application	PDX model	Time point	Tumor size
H&E stainings	PDX5474	42	401
H&E stainings	PDX4582	77	94
H&E stainings	PDX1002	38	55
H&E stainings	PDX1004	78	413
H&E stainings	PDX9228	36	256
Flowcytometry	PDX5474	55	405
Flowcytometry	PDX5474	55	372
Flowcytometry	PDX4582	144	340
Flowcytometry	PDX1002	61	214
Flowcytometry	PDX1004	67	388
Flowcytometry	PDX1004	67	341
Flowcytometry	PDX9228	45	305
Flowcytometry	PDX9228	45	312
Immunofluorescence	PDX5474	52	388
Immunofluorescence	PDX5474	52	321
Immunofluorescence	PDX5474	42	401
Immunofluorescence	PDX5474	42	377
Immunofluorescence	PDX5474	42	381
Immunofluorescence	PDX4582	77	294
Immunofluorescence	PDX4582	41	196
Immunofluorescence	PDX4582	33	96
Immunofluorescence	PDX4582	33	213
Immunofluorescence	PDX1002	38	50,8
Immunofluorescence	PDX1002	38	55,1
Immunofluorescence	PDX1002	38	51,3
Immunofluorescence	PDX1002	38	54,6
Immunofluorescence	PDX1002	38	82,8
Immunofluorescence	PDX1004	78	504
Immunofluorescence	PDX1004	78	413
Immunofluorescence	PDX1004	74	643
Immunofluorescence	PDX9228	39	384
Immunofluorescence	PDX9228	36	256
Immunofluorescence	PDX9228	35	496
Immunofluorescence	PDX9228	35	384
Immunofluorescence	PDX9228	35	531
Immunofluorescence	PDX9228	35	605
Immunofluorescence	PDX9228	42	288

Supplementary table S1. *PDX tumor size and time points for used PDX tumors in different applications. Tumor size is measured as Width x Width x Length x 0.5. Timepoints represents time from transplantation of PDX tissue to excision of tumor tissue*
